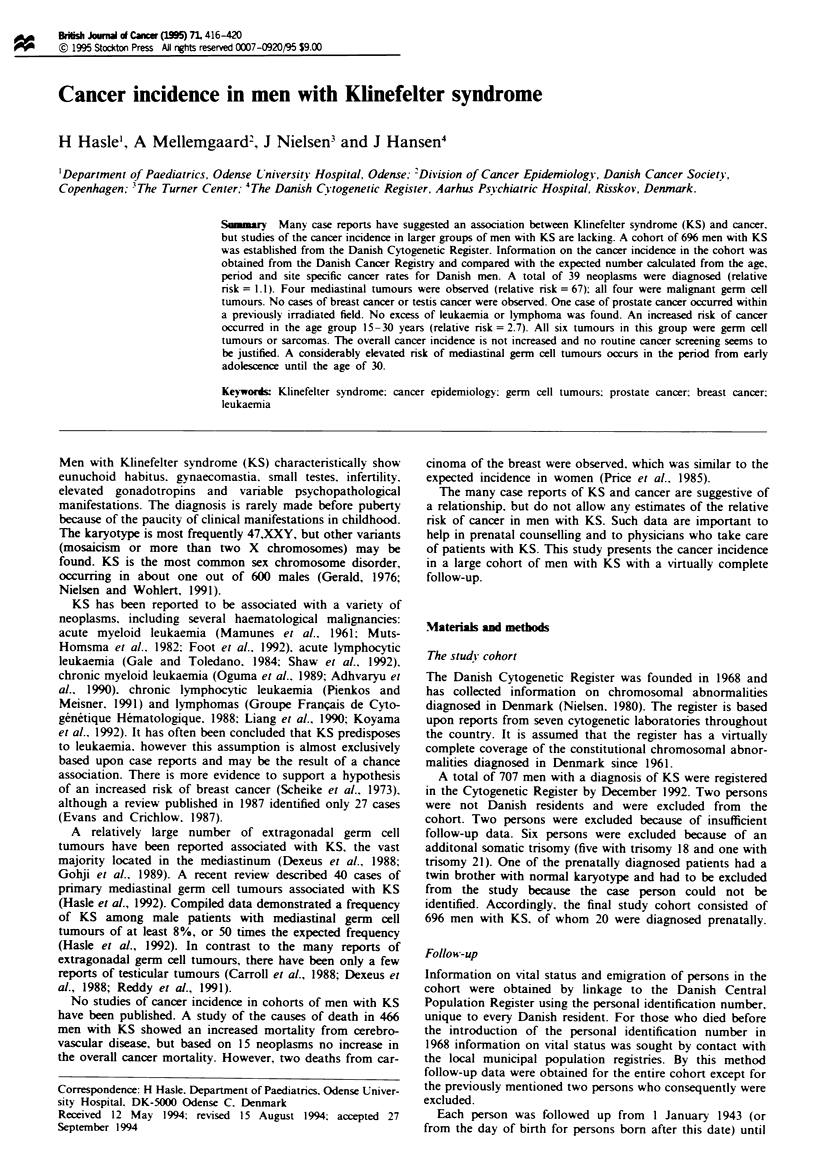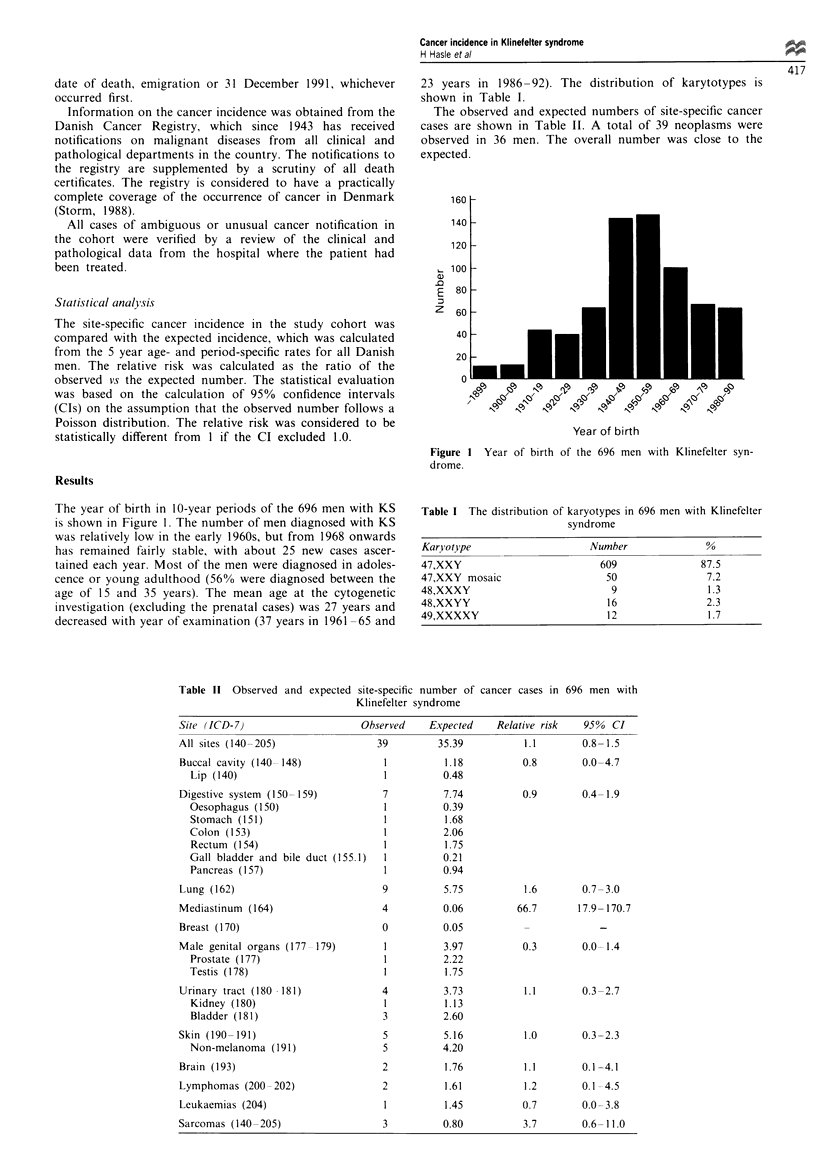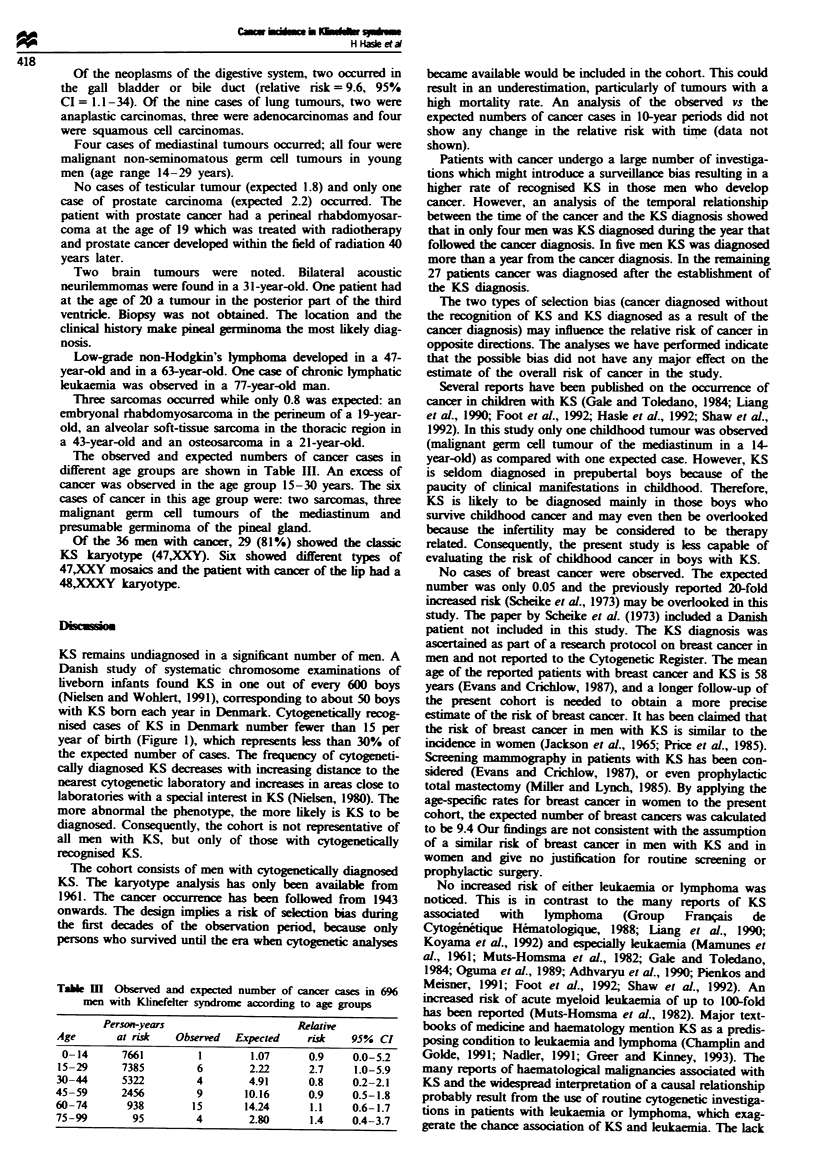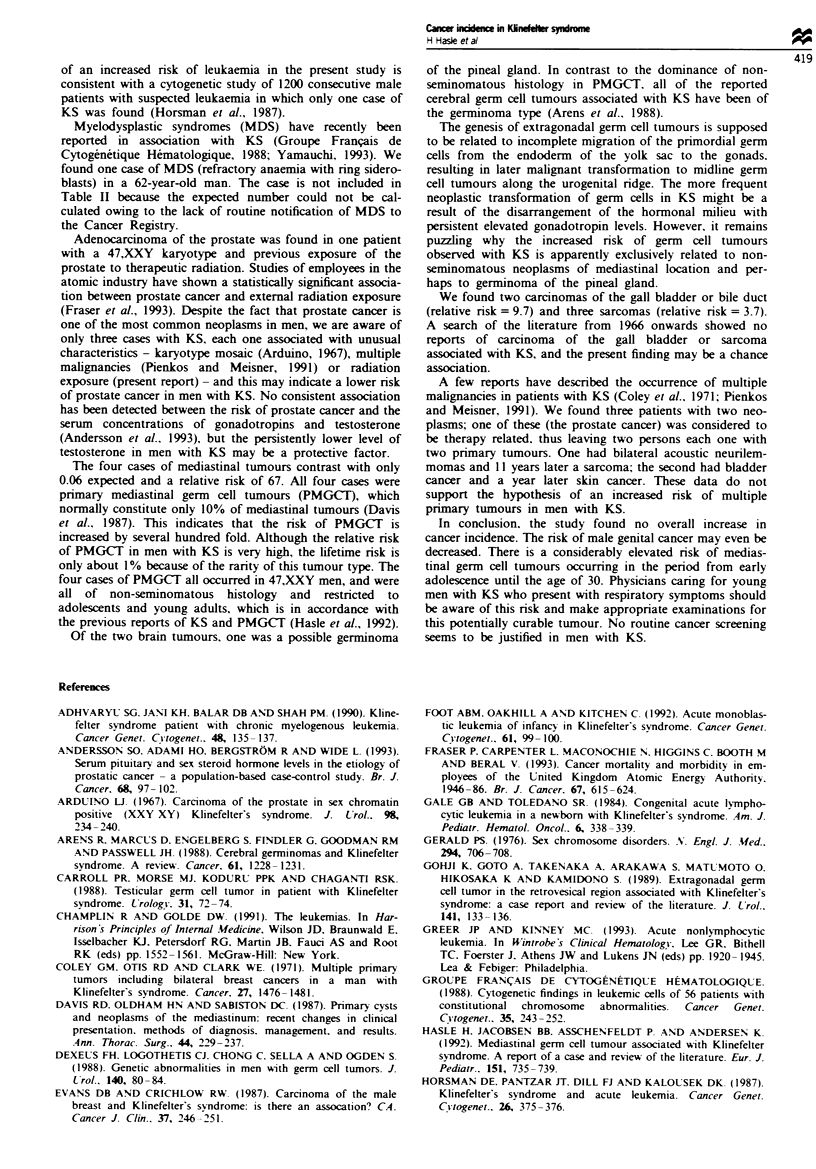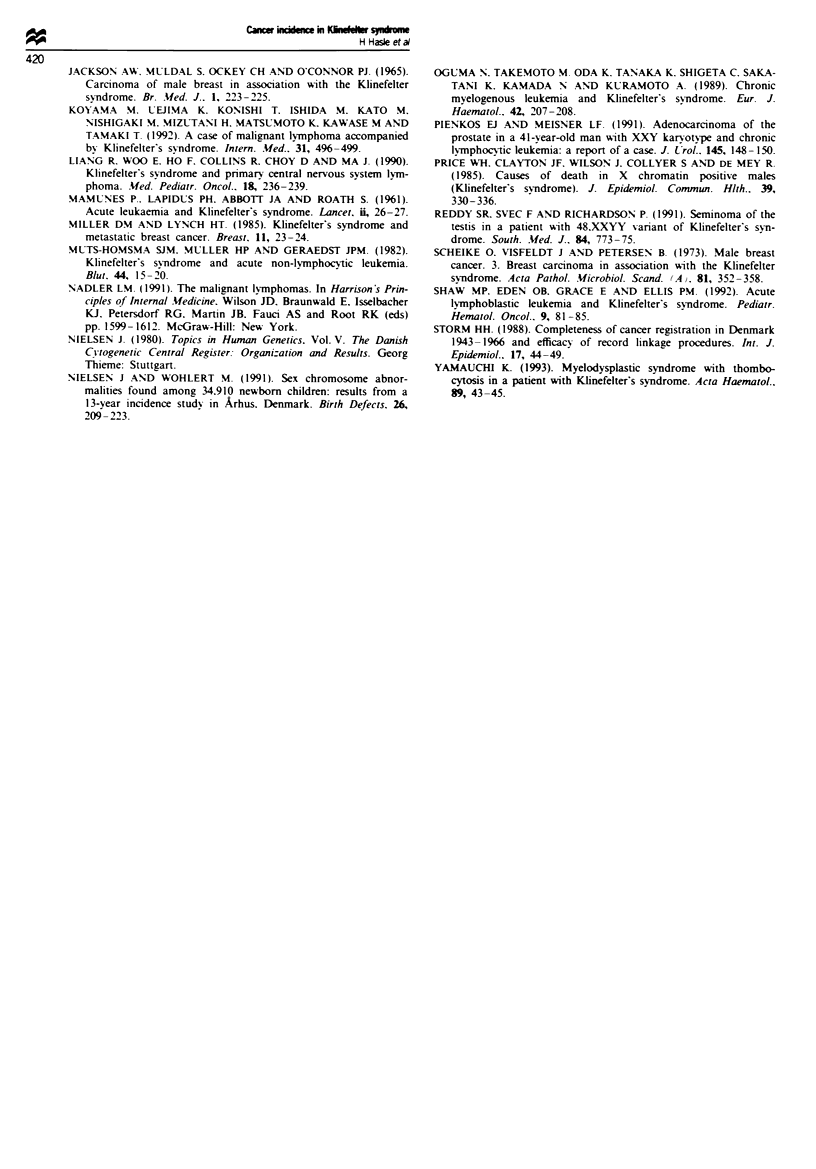# Cancer incidence in men with Klinefelter syndrome.

**DOI:** 10.1038/bjc.1995.85

**Published:** 1995-02

**Authors:** H. Hasle, A. Mellemgaard, J. Nielsen, J. Hansen

**Affiliations:** Department of Paediatrics, Odense University Hospital, Denmark.

## Abstract

Many case reports have suggested an association between Klinefelter syndrome (KS) and cancer, but studies of the cancer incidence in larger groups of men with KS are lacking. A cohort of 696 men with KS was established from the Danish Cytogenetic Register. Information on the cancer incidence in the cohort was obtained from the Danish Cancer Registry and compared with the expected number calculated from the age, period and site specific cancer rates for Danish men. A total of 39 neoplasms were diagnosed (relative risk = 1.1). Four mediastinal tumours were observed (relative risk = 67); all four were malignant germ cell tumours. No cases of breast cancer or testis cancer were observed. One case of prostate cancer occurred within a previously irradiated field. No excess of leukaemia or lymphoma was found. An increased risk of cancer occurred in the age group 15-30 years (relative risk = 2.7). All six tumours in this group were germ cell tumours or sarcomas. The overall cancer incidence is not increased and no routine cancer screening seems to be justified. A considerably elevated risk of mediastinal germ cell tumours occurs in the period from early adolescence until the age of 30.


British Journal of Cancer (1995) 71, 416-420

© 1995 Stockton Press All rights reserved 0007-0920/95 $9.00

### Cancer incidence in men with Klinefelter syndrome

H Hasle[1], A Mellemgaard[2], J Nielsen[3] and J Hansen[4]

[1]Department of Paediatrics, Odense University Hospital, Odense; [2]Division of Cancer Epidemiology, Danish Cancer Society, Copenhagen; [3]The Turner Center; [4]The Danish Cytogenetic Register, Aarhus Psychiatric Hospital, Risskov, Denmark.

**Summary** Many case reports have suggested an association between Klinefelter syndrome (KS) and cancer, but studies of the cancer incidence in larger groups of men with KS are lacking. A cohort of 696 men with KS was established from the Danish Cytogenetic Register. Information on the cancer incidence in the cohort was obtained from the Danish Cancer Registry and compared with the expected number calculated from the age, period and site specific cancer rates for Danish men. A total of 39 neoplasms were diagnosed (relative risk = 1.1). Four mediastinal tumours were observed (relative risk = 67); all four were malignant germ cell tumours. No cases of breast cancer or testis cancer were observed. One case of prostate cancer occurred within a previously irradiated field. No excess of leukaemia or lymphoma was found. An increased risk of cancer occurred in the age group 15–30 years (relative risk = 2.7). All six tumours in this group were germ cell tumours or sarcomas. The overall cancer incidence is not increased and no routine cancer screening seems to be justified. A considerably elevated risk of mediastinal germ cell tumours occurs in the period from early adolescence until the age of 30.

**Keywords:** Klinefelter syndrome; cancer epidemiology; germ cell tumours; prostate cancer; breast cancer; leukaemia

Men with Klinefelter syndrome (KS) characteristically show eunuchoid habitus, gynaecomastia, small testes, infertility, elevated gonadotropins and variable psychopathological manifestations. The diagnosis is rarely made before puberty because of the paucity of clinical manifestations in childhood. The karyotype is most frequently 47,XXY, but other variants (mosaicism or more than two X chromosomes) may be found. KS is the most common sex chromosome disorder, occurring in about one out of 600 males (Gerald, 1976; Nielsen and Wohlert, 1991).

KS has been reported to be associated with a variety of neoplasms, including several haematological malignancies: acute myeloid leukaemia (Mamunes *et al.*, 1961; Muts-Homsma *et al.*, 1982; Foot *et al.*, 1992), acute lymphocytic leukaemia (Gale and Toledano, 1984; Shaw *et al.*, 1992), chronic myeloid leukaemia (Oguma *et al.*, 1989; Adhvaryu *et al.*, 1990), chronic lymphocytic leukaemia (Pienkos and Meisner, 1991) and lymphomas (Groupe Français de Cytogénétique Hématologique, 1988; Liang *et al.*, 1990; Koyama *et al.*, 1992). It has often been concluded that KS predisposes to leukaemia, however this assumption is almost exclusively based upon case reports and may be the result of a chance association. There is more evidence to support a hypothesis of an increased risk of breast cancer (Scheike *et al.*, 1973), although a review published in 1987 identified only 27 cases (Evans and Crichlow, 1987).

A relatively large number of extragonadal germ cell tumours have been reported associated with KS, the vast majority located in the mediastinum (Dexeus *et al.*, 1988; Gohji *et al.*, 1989). A recent review described 40 cases of primary mediastinal germ cell tumours associated with KS (Hasle *et al.*, 1992). Compiled data demonstrated a frequency of KS among male patients with mediastinal germ cell tumours of at least 8%, or 50 times the expected frequency (Hasle *et al.*, 1992). In contrast to the many reports of extragonadal germ cell tumours, there have been only a few reports of testicular tumours (Carroll *et al.*, 1988; Dexeus *et al.*, 1988; Reddy *et al.*, 1991).

No studies of cancer incidence in cohorts of men with KS have been published. A study of the causes of death in 466 men with KS showed an increased mortality from cerebrovascular disease, but based on 15 neoplasms no increase in the overall cancer mortality. However, two deaths from carcinoma of the breast were observed, which was similar to the expected incidence in women (Price *et al.*, 1985).

The many case reports of KS and cancer are suggestive of a relationship, but do not allow any estimates of the relative risk of cancer in men with KS. Such data are important to help in prenatal counselling and to physicians who take care of patients with KS. This study presents the cancer incidence in a large cohort of men with KS with a virtually complete follow-up.

### Materials and methods

### *The study cohort*

The Danish Cytogenetic Register was founded in 1968 and has collected information on chromosomal abnormalities diagnosed in Denmark (Nielsen, 1980). The register is based upon reports from seven cytogenetic laboratories throughout the country. It is assumed that the register has a virtually complete coverage of the constitutional chromosomal abnormalities diagnosed in Denmark since 1961.

A total of 707 men with a diagnosis of KS were registered in the Cytogenetic Register by December 1992. Two persons were not Danish residents and were excluded from the cohort. Two persons were excluded because of insufficient follow-up data. Six persons were excluded because of an additonal somatic trisomy (five with trisomy 18 and one with trisomy 21). One of the prenatally diagnosed patients had a twin brother with normal karyotype and had to be excluded from the study because the case person could not be identified. Accordingly, the final study cohort consisted of 696 men with KS, of whom 20 were diagnosed prenatally.

### *Follow-up*

Information on vital status and emigration of persons in the cohort were obtained by linkage to the Danish Central Population Register using the personal identification number, unique to every Danish resident. For those who died before the introduction of the personal identification number in 1968 information on vital status was sought by contact with the local municipal population registries. By this method follow-up data were obtained for the entire cohort except for the previously mentioned two persons who consequently were excluded.

Each person was followed up from 1 January 1943 (or from the day of birth for persons born after this date) until

Correspondence: H Hasle, Department of Paediatrics, Odense University Hospital, DK-5000 Odense C, Denmark
Received 12 May 1994; revised 15 August 1994; accepted 27 September 1994

date of death, emigration or 31 December 1991, whichever occurred first.

Information on the cancer incidence was obtained from the Danish Cancer Registry, which since 1943 has received notifications on malignant diseases from all clinical and pathological departments in the country. The notifications to the registry are supplemented by a scrutiny of all death certificates. The registry is considered to have a practically complete coverage of the occurrence of cancer in Denmark (Storm, 1988).

All cases of ambiguous or unusual cancer notification in the cohort were verified by a review of the clinical and pathological data from the hospital where the patient had been treated.

*Statistical analysis*

The site-specific cancer incidence in the study cohort was compared with the expected incidence, which was calculated from the 5 year age- and period-specific rates for all Danish men. The relative risk was calculated as the ratio of the observed *vs* the expected number. The statistical evaluation was based on the calculation of 95% confidence intervals (CIs) on the assumption that the observed number follows a Poisson distribution. The relative risk was considered to be statistically different from 1 if the CI excluded 1.0.

### Results

The year of birth in 10-year periods of the 696 men with KS is shown in Figure 1. The number of men diagnosed with KS was relatively low in the early 1960s, but from 1968 onwards has remained fairly stable, with about 25 new cases ascertained each year. Most of the men were diagnosed in adolescence or young adulthood (56% were diagnosed between the age of 15 and 35 years). The mean age at the cytogenetic investigation (excluding the prenatal cases) was 27 years and decreased with year of examination (37 years in 1961–65 and 23 years in 1986–92). The distribution of karytotypes is shown in Table I.

The observed and expected numbers of site-specific cancer cases are shown in Table II. A total of 39 neoplasms were observed in 36 men. The overall number was close to the expected.

**Figure 1** Year of birth of the 696 men with Klinefelter syndrome.

**Table I** The distribution of karyotypes in 696 men with Klinefelter syndrome

| *Karyotype* | *Number* | *%* |
|---|---|---|
| 47,XXY | 609 | 87.5 |
| 47,XXY mosaic | 50 | 7.2 |
| 48,XXXY | 9 | 1.3 |
| 48,XXYY | 16 | 2.3 |
| 49,XXXXY | 12 | 1.7 |

**Table II** Observed and expected site-specific number of cancer cases in 696 men with Klinefelter syndrome

| *Site (ICD-7)* | *Observed* | *Expected* | *Relative risk* | *95% CI* |
|---|---|---|---|---|
| All sites (140–205) | 39 | 35.39 | 1.1 | 0.8–1.5 |
| Buccal cavity (140–148) | 1 | 1.18 | 0.8 | 0.0–4.7 |
| Lip (140) | 1 | 0.48 | | |
| Digestive system (150–159) | 7 | 7.74 | 0.9 | 0.4–1.9 |
| Oesophagus (150) | 1 | 0.39 | | |
| Stomach (151) | 1 | 1.68 | | |
| Colon (153) | 1 | 2.06 | | |
| Rectum (154) | 1 | 1.75 | | |
| Gall bladder and bile duct (155.1) | 1 | 0.21 | | |
| Pancreas (157) | 1 | 0.94 | | |
| Lung (162) | 9 | 5.75 | 1.6 | 0.7–3.0 |
| Mediastinum (164) | 4 | 0.06 | 66.7 | 17.9–170.7 |
| Breast (170) | 0 | 0.05 | – | – |
| Male genital organs (177–179) | 1 | 3.97 | 0.3 | 0.0–1.4 |
| Prostate (177) | 1 | 2.22 | | |
| Testis (178) | 1 | 1.75 | | |
| Urinary tract (180–181) | 4 | 3.73 | 1.1 | 0.3–2.7 |
| Kidney (180) | 1 | 1.13 | | |
| Bladder (181) | 3 | 2.60 | | |
| Skin (190–191) | 5 | 5.16 | 1.0 | 0.3–2.3 |
| Non-melanoma (191) | 5 | 4.20 | | |
| Brain (193) | 2 | 1.76 | 1.1 | 0.1–4.1 |
| Lymphomas (200–202) | 2 | 1.61 | 1.2 | 0.1–4.5 |
| Leukaemias (204) | 1 | 1.45 | 0.7 | 0.0–3.8 |
| Sarcomas (140–205) | 3 | 0.80 | 3.7 | 0.6–11.0 |

Of the neoplasms of the digestive system, two occurred in the gall bladder or bile duct (relative risk = 9.6, 95% CI = 1.1-34). Of the nine cases of lung tumours, two were anaplastic carcinomas, three were adenocarcinomas and four were squamous cell carcinomas.

Four cases of mediastinal tumours occurred; all four were malignant non-seminomatous germ cell tumours in young men (age range 14-29 years).

No cases of testicular tumour (expected 1.8) and only one case of prostate carcinoma (expected 2.2) occurred. The patient with prostate cancer had a perineal rhabdomyosarcoma at the age of 19 which was treated with radiotherapy and prostate cancer developed within the field of radiation 40 years later.

Two brain tumours were noted. Bilateral acoustic neurilemmomas were found in a 31-year-old. One patient had at the age of 20 a tumour in the posterior part of the third ventricle. Biopsy was not obtained. The location and the clinical history make pineal germinoma the most likely diagnosis.

Low-grade non-Hodgkin's lymphoma developed in a 47-year-old and in a 63-year-old. One case of chronic lymphatic leukaemia was observed in a 77-year-old man.

Three sarcomas occurred while only 0.8 was expected: an embryonal rhabdomyosarcoma in the perineum of a 19-year-old, an alveolar soft-tissue sarcoma in the thoracic region in a 43-year-old and an osteosarcoma in a 21-year-old.

The observed and expected numbers of cancer cases in different age groups are shown in Table III. An excess of cancer was observed in the age group 15-30 years. The six cases of cancer in this age group were: two sarcomas, three malignant germ cell tumours of the mediastinum and presumable germinoma of the pineal gland.

Of the 36 men with cancer, 29 (81%) showed the classic KS karyotype (47,XXY). Six showed different types of 47,XXY mosaics and the patient with cancer of the lip had a 48,XXXY karyotype.

### Discussion

KS remains undiagnosed in a significant number of men. A Danish study of systematic chromosome examinations of liveborn infants found KS in one out of every 600 boys (Nielsen and Wohlert, 1991), corresponding to about 50 boys with KS born each year in Denmark. Cytogenetically recognised cases of KS in Denmark number fewer than 15 per year of birth (Figure 1), which represents less than 30% of the expected number of cases. The frequency of cytogenetically diagnosed KS decreases with increasing distance to the nearest cytogenetic laboratory and increases in areas close to laboratories with a special interest in KS (Nielsen, 1980). The more abnormal the phenotype, the more likely is KS to be diagnosed. Consequently, the cohort is not representative of all men with KS, but only of those with cytogenetically recognised KS.

The cohort consists of men with cytogenetically diagnosed KS. The karyotype analysis has only been available from 1961. The cancer occurrence has been followed from 1943 onwards. The design implies a risk of selection bias during the first decades of the observation period, because only persons who survived until the era when cytogenetic analyses became available would be included in the cohort. This could result in an underestimation, particularly of tumours with a high mortality rate. An analysis of the observed *vs* the expected numbers of cancer cases in 10-year periods did not show any change in the relative risk with time (data not shown).

Patients with cancer undergo a large number of investigations which might introduce a surveillance bias resulting in a higher rate of recognised KS in those men who develop cancer. However, an analysis of the temporal relationship between the time of the cancer and the KS diagnosis showed that in only four men was KS diagnosed during the year that followed the cancer diagnosis. In five men KS was diagnosed more than a year from the cancer diagnosis. In the remaining 27 patients cancer was diagnosed after the establishment of the KS diagnosis.

The two types of selection bias (cancer diagnosed without the recognition of KS and KS diagnosed as a result of the cancer diagnosis) may influence the relative risk of cancer in opposite directions. The analyses we have performed indicate that the possible bias did not have any major effect on the estimate of the overall risk of cancer in the study.

Several reports have been published on the occurrence of cancer in children with KS (Gale and Toledano, 1984; Liang *et al.*, 1990; Foot *et al.*, 1992; Hasle *et al.*, 1992; Shaw *et al.*, 1992). In this study only one childhood tumour was observed (malignant germ cell tumour of the mediastinum in a 14-year-old) as compared with one expected case. However, KS is seldom diagnosed in prepubertal boys because of the paucity of clinical manifestations in childhood. Therefore, KS is likely to be diagnosed mainly in those boys who survive childhood cancer and may even then be overlooked because the infertility may be considered to be therapy related. Consequently, the present study is less capable of evaluating the risk of childhood cancer in boys with KS.

No cases of breast cancer were observed. The expected number was only 0.05 and the previously reported 20-fold increased risk (Scheike *et al.*, 1973) may be overlooked in this study. The paper by Scheike *et al.* (1973) included a Danish patient not included in this study. The KS diagnosis was ascertained as part of a research protocol on breast cancer in men and not reported to the Cytogenetic Register. The mean age of the reported patients with breast cancer and KS is 58 years (Evans and Crichlow, 1987), and a longer follow-up of the present cohort is needed to obtain a more precise estimate of the risk of breast cancer. It has been claimed that the risk of breast cancer in men with KS is similar to the incidence in women (Jackson *et al.*, 1965; Price *et al.*, 1985). Screening mammography in patients with KS has been considered (Evans and Crichlow, 1987), or even prophylactic total mastectomy (Miller and Lynch, 1985). By applying the age-specific rates for breast cancer in women to the present cohort, the expected number of breast cancers was calculated to be 9.4 Our findings are not consistent with the assumption of a similar risk of breast cancer in men with KS and in women and give no justification for routine screening or prophylactic surgery.

No increased risk of either leukaemia or lymphoma was noticed. This is in contrast to the many reports of KS associated with lymphoma (Group Français de Cytogénétique Hématologique, 1988; Liang *et al.*, 1990; Koyama *et al.*, 1992) and especially leukaemia (Mamunes *et al.*, 1961; Muts-Homsma *et al.*, 1982; Gale and Toledano, 1984; Oguma *et al.*, 1989; Adhvaryu *et al.*, 1990; Pienkos and Meisner, 1991; Foot *et al.*, 1992; Shaw *et al.*, 1992). An increased risk of acute myeloid leukaemia of up to 100-fold has been reported (Muts-Homsma *et al.*, 1982). Major textbooks of medicine and haematology mention KS as a predisposing condition to leukaemia and lymphoma (Champlin and Golde, 1991; Nadler, 1991; Greer and Kinney, 1993). The many reports of haematological malignancies associated with KS and the widespread interpretation of a causal relationship probably result from the use of routine cytogenetic investigations in patients with leukaemia or lymphoma, which exaggerate the chance association of KS and leukaemia. The lack

**Table III** Observed and expected number of cancer cases in 696 men with Klinefelter syndrome according to age groups

| *Age* | *Person-years at risk* | *Observed* | *Expected* | *Relative risk* | *95% CI* |
|---|---|---|---|---|---|
| 0-14 | 7661 | 1 | 1.07 | 0.9 | 0.0-5.2 |
| 15-29 | 7385 | 6 | 2.22 | 2.7 | 1.0-5.9 |
| 30-44 | 5322 | 4 | 4.91 | 0.8 | 0.2-2.1 |
| 45-59 | 2456 | 9 | 10.16 | 0.9 | 0.5-1.8 |
| 60-74 | 938 | 15 | 14.24 | 1.1 | 0.6-1.7 |
| 75-99 | 95 | 4 | 2.80 | 1.4 | 0.4-3.7 |

of an increased risk of leukaemia in the present study is consistent with a cytogenetic study of 1200 consecutive male patients with suspected leukaemia in which only one case of KS was found (Horsman *et al*., 1987).

Myelodysplastic syndromes (MDS) have recently been reported in association with KS (Groupe Français de Cytogénétique Hématologique, 1988; Yamauchi, 1993). We found one case of MDS (refractory anaemia with ring sideroblasts) in a 62-year-old man. The case is not included in Table II because the expected number could not be calculated owing to the lack of routine notification of MDS to the Cancer Registry.

Adenocarcinoma of the prostate was found in one patient with a 47,XXY karyotype and previous exposure of the prostate to therapeutic radiation. Studies of employees in the atomic industry have shown a statistically significant association between prostate cancer and external radiation exposure (Fraser *et al*., 1993). Despite the fact that prostate cancer is one of the most common neoplasms in men, we are aware of only three cases with KS, each one associated with unusual characteristics – karyotype mosaic (Arduino, 1967), multiple malignancies (Pienkos and Meisner, 1991) or radiation exposure (present report) – and this may indicate a lower risk of prostate cancer in men with KS. No consistent association has been detected between the risk of prostate cancer and the serum concentrations of gonadotropins and testosterone (Andersson *et al*., 1993), but the persistently lower level of testosterone in men with KS may be a protective factor.

The four cases of mediastinal tumours contrast with only 0.06 expected and a relative risk of 67. All four cases were primary mediastinal germ cell tumours (PMGCT), which normally constitute only 10% of mediastinal tumours (Davis *et al*., 1987). This indicates that the risk of PMGCT is increased by several hundred fold. Although the relative risk of PMGCT in men with KS is very high, the lifetime risk is only about 1% because of the rarity of this tumour type. The four cases of PMGCT all occurred in 47,XXY men, and were all of non-seminomatous histology and restricted to adolescents and young adults, which is in accordance with the previous reports of KS and PMGCT (Hasle *et al*., 1992).

Of the two brain tumours, one was a possible germinoma of the pineal gland. In contrast to the dominance of non-seminomatous histology in PMGCT, all of the reported cerebral germ cell tumours associated with KS have been of the germinoma type (Arens *et al*., 1988).

The genesis of extragonadal germ cell tumours is supposed to be related to incomplete migration of the primordial germ cells from the endoderm of the yolk sac to the gonads, resulting in later malignant transformation to midline germ cell tumours along the urogenital ridge. The more frequent neoplastic transformation of germ cells in KS might be a result of the disarrangement of the hormonal milieu with persistent elevated gonadotropin levels. However, it remains puzzling why the increased risk of germ cell tumours observed with KS is apparently exclusively related to non-seminomatous neoplasms of mediastinal location and perhaps to germinoma of the pineal gland.

We found two carcinomas of the gall bladder or bile duct (relative risk = 9.7) and three sarcomas (relative risk = 3.7). A search of the literature from 1966 onwards showed no reports of carcinoma of the gall bladder or sarcoma associated with KS, and the present finding may be a chance association.

A few reports have described the occurrence of multiple malignancies in patients with KS (Coley *et al*., 1971; Pienkos and Meisner, 1991). We found three patients with two neoplasms; one of these (the prostate cancer) was considered to be therapy related, thus leaving two persons each one with two primary tumours. One had bilateral acoustic neurilemmomas and 11 years later a sarcoma; the second had bladder cancer and a year later skin cancer. These data do not support the hypothesis of an increased risk of multiple primary tumours in men with KS.

In conclusion, the study found no overall increase in cancer incidence. The risk of male genital cancer may even be decreased. There is a considerably elevated risk of mediastinal germ cell tumours occurring in the period from early adolescence until the age of 30. Physicians caring for young men with KS who present with respiratory symptoms should be aware of this risk and make appropriate examinations for this potentially curable tumour. No routine cancer screening seems to be justified in men with KS.

### References

ADHVARYU SG, JANI KH, BALAR DB AND SHAH PM. (1990). Klinefelter syndrome patient with chronic myelogenous leukemia. *Cancer Genet. Cytogenet.*, **48**, 135–137.

ANDERSSON SO, ADAMI HO, BERGSTRÖM R AND WIDE L. (1993). Serum pituitary and sex steroid hormone levels in the etiology of prostatic cancer – a population-based case-control study. *Br. J. Cancer*, **68**, 97–102.

ARDUINO LJ. (1967). Carcinoma of the prostate in sex chromatin positive (XXY XY) Klinefelter's syndrome. *J. Urol.*, **98**, 234–240.

ARENS R, MARCUS D, ENGELBERG S, FINDLER G, GOODMAN RM AND PASSWELL JH. (1988). Cerebral germinomas and Klinefelter syndrome. A review. *Cancer*, **61**, 1228–1231.

CARROLL PR, MORSE MJ, KODURU PPK AND CHAGANTI RSK. (1988). Testicular germ cell tumor in patient with Klinefelter syndrome. *Urology*, **31**, 72–74.

CHAMPLIN R AND GOLDE DW. (1991). The leukemias. In *Harrison's Principles of Internal Medicine*, Wilson JD, Braunwald E, Isselbacher KJ, Petersdorf RG, Martin JB, Fauci AS and Root RK (eds) pp. 1552–1561. McGraw-Hill: New York.

COLEY GM, OTIS RD AND CLARK WE. (1971). Multiple primary tumors including bilateral breast cancers in a man with Klinefelter's syndrome. *Cancer*, **27**, 1476–1481.

DAVIS RD, OLDHAM HN AND SABISTON DC. (1987). Primary cysts and neoplasms of the mediastinum: recent changes in clinical presentation, methods of diagnosis, management, and results. *Ann. Thorac. Surg.*, **44**, 229–237.

DEXEUS FH, LOGOTHETIS CJ, CHONG C, SELLA A AND OGDEN S. (1988). Genetic abnormalities in men with germ cell tumors. *J. Urol.*, **140**, 80–84.

EVANS DB AND CRICHLOW RW. (1987). Carcinoma of the male breast and Klinefelter's syndrome: is there an assocation? *CA. Cancer J. Clin.*, **37**, 246–251.

FOOT ABM, OAKHILL A AND KITCHEN C. (1992). Acute monoblastic leukemia of infancy in Klinefelter's syndrome. *Cancer Genet. Cytogenet.*, **61**, 99–100.

FRASER P, CARPENTER L, MACONOCHIE N, HIGGINS C, BOOTH M AND BERAL V. (1993). Cancer mortality and morbidity in employees of the United Kingdom Atomic Energy Authority, 1946–86. *Br. J. Cancer*, **67**, 615–624.

GALE GB AND TOLEDANO SR. (1984). Congenital acute lymphocytic leukemia in a newborn with Klinefelter's syndrome. *Am. J. Pediatr. Hematol. Oncol.*, **6**, 338–339.

GERALD PS. (1976). Sex chromosome disorders. *N. Engl. J. Med.*, **294**, 706–708.

GOHJI K, GOTO A, TAKENAKA A, ARAKAWA S, MATUMOTO O, HIKOSAKA K AND KAMIDONO S. (1989). Extragonadal germ cell tumor in the retrovesical region associated with Klinefelter's syndrome: a case report and review of the literature. *J. Urol.*, **141**, 133–136.

GREER JP AND KINNEY MC. (1993). Acute nonlymphocytic leukemia. In *Wintrobe's Clinical Hematology*, Lee GR, Bithell TC, Foerster J, Athens JW and Lukens JN (eds) pp. 1920–1945. Lea & Febiger: Philadelphia.

GROUPE FRANÇAIS DE CYTOGÉNÉTIQUE HÉMATOLOGIQUE. (1988). Cytogenetic findings in leukemic cells of 56 patients with constitutional chromosome abnormalities. *Cancer Genet. Cytogenet.*, **35**, 243–252.

HASLE H, JACOBSEN BB, ASSCHENFELDT P AND ANDERSEN K. (1992). Mediastinal germ cell tumour associated with Klinefelter syndrome. A report of a case and review of the literature. *Eur. J. Pediatr.*, **151**, 735–739.

HORSMAN DE, PANTZAR JT, DILL FJ AND KALOUSEK DK. (1987). Klinefelter's syndrome and acute leukemia. *Cancer Genet. Cytogenet.*, **26**, 375–376.

JACKSON AW, MULDAL S, OCKEY CH AND O'CONNOR PJ. (1965). Carcinoma of male breast in association with the Klinefelter syndrome. *Br. Med. J.*, **1**, 223-225.

KOYAMA M, UEJIMA K, KONISHI T, ISHIDA M, KATO M, NISHIGAKI M, MIZUTANI H, MATSUMOTO K, KAWASE M AND TAMAKI T. (1992). A case of malignant lymphoma accompanied by Klinefelter's syndrome. *Intern. Med.*, **31**, 496-499.

LIANG R, WOO E, HO F, COLLINS R, CHOY D AND MA J. (1990). Klinefelter's syndrome and primary central nervous system lymphoma. *Med. Pediatr. Oncol.*, **18**, 236-239.

MAMUNES P., LAPIDUS PH, ABBOTT JA AND ROATH S. (1961). Acute leukaemia and Klinefelter's syndrome. *Lancet*, **ii**, 26-27.

MILLER DM AND LYNCH HT. (1985). Klinefelter's syndrome and metastatic breast cancer. *Breast*, **11**, 23-24.

MUTS-HOMSMA SJM, MULLER HP AND GERAEDST JPM. (1982). Klinefelter's syndrome and acute non-lymphocytic leukemia. *Blut*, **44**, 15-20.

NADLER LM. (1991). The malignant lymphomas. In *Harrison's Principles of Internal Medicine*, Wilson JD, Braunwald E, Isselbacher KJ, Petersdorf RG, Martin JB, Fauci AS and Root RK (eds) pp. 1599-1612. McGraw-Hill: New York.

NIELSEN J. (1980). *Topics in Human Genetics*, Vol. V. *The Danish Cytogenetic Central Register: Organization and Results*. Georg Thieme: Stuttgart.

NIELSEN J AND WOHLERT M. (1991). Sex chromosome abnormalities found among 34.910 newborn children: results from a 13-year incidence study in Århus, Denmark. *Birth Defects*, **26**, 209-223.

OGUMA N, TAKEMOTO M, ODA K, TANAKA K, SHIGETA C, SAKATANI K, KAMADA N AND KURAMOTO A. (1989). Chronic myelogenous leukemia and Klinefelter's syndrome. *Eur. J. Haematol.*, **42**, 207-208.

PIENKOS EJ AND MEISNER LF. (1991). Adenocarcinoma of the prostate in a 41-year-old man with XXY karyotype and chronic lymphocytic leukemia: a report of a case. *J. Urol.*, **145**, 148-150.

PRICE WH, CLAYTON JF, WILSON J, COLLYER S AND DE MEY R. (1985). Causes of death in X chromatin positive males (Klinefelter's syndrome). *J. Epidemiol. Commun. Hlth.*, **39**, 330-336.

REDDY SR, SVEC F AND RICHARDSON P. (1991). Seminoma of the testis in a patient with 48,XXYY variant of Klinefelter's syndrome. *South. Med. J.*, **84**, 773-75.

SCHEIKE O, VISFELDT J AND PETERSEN B. (1973). Male breast cancer. 3. Breast carcinoma in association with the Klinefelter syndrome. *Acta Pathol. Microbiol. Scand. (A)*, **81**, 352-358.

SHAW MP, EDEN OB, GRACE E AND ELLIS PM. (1992). Acute lymphoblastic leukemia and Klinefelter's syndrome. *Pediatr. Hematol. Oncol.*, **9**, 81-85.

STORM HH. (1988). Completeness of cancer registration in Denmark 1943-1966 and efficacy of record linkage procedures. *Int. J. Epidemiol.*, **17**, 44-49.

YAMAUCHI K. (1993). Myelodysplastic syndrome with thombocytosis in a patient with Klinefelter's syndrome. *Acta Haematol.*, **89**, 43-45.